# Minimally invasive dynamic screw stabilization using cortical bone trajectory

**DOI:** 10.1186/s12891-020-03629-z

**Published:** 2020-09-10

**Authors:** Chih-Chang Chang, Chao-Hung Kuo, Hsuan-Kan Chang, Tsung-Hsi Tu, Li-Yu Fay, Jau-Ching Wu, Henrich Cheng, Wen-Cheng Huang

**Affiliations:** 1 Department of Neurosurgery, Neurological Institute, Taipei Veterans General Hospital, Room 525, 17F, No. 201, Shih-Pai Road, Sec. 2, Beitou, Taipei, 11217 Taiwan; 2 School of Medicine, National Yang-Ming University, Taipei, Taiwan; 3 Department of BioMedical Engineering, National Yang-Ming University, Taipei, Taiwan; 4 Institute of Pharmacology, National Yang-Ming University, Taipei, Taiwan

**Keywords:** Minimally invasive spine surgery, Spondylolisthesis, Dynamic stabilization, Cortical bone trajectory (CBT)

## Abstract

**Background:**

The conventional pedicle-screw-based dynamic stabilization process involves dissection of the Wiltse plane to cannulate the pedicles, which cannot be undertaken with minimal surgical invasion. Despite some reports having demonstrated satisfactory outcomes of dynamic stabilization in the management of low-grade spondylolisthesis, the extensive soft tissue dissection involved during pedicle screw insertion substantially compromises the designed rationale of motion (muscular) preservation. The authors report on a novel method for minimally invasive insertion of dynamic screws and a mini case series.

**Methods:**

The authors describe innovations for inserting dynamic screws via the cortical bone trajectory (CBT) under spinal navigation. All the detailed surgical procedures and clinical data are demonstrated.

**Results:**

A total of four (2 females) patients (mean age 64.75 years) with spinal stenosis at L4–5 were included. By a combination of microscopic decompression and image-guided CBT screw insertion, laminectomy and dynamic screw stabilization were achieved via one small skin incision (less than 3 cm). These patients’ back and leg pain improved significantly after the surgery.

**Conclusion:**

This innovative dynamic screw stabilization via the CBT involved no discectomy (or removal of sequestrated fragment only), no interbody fusion, and little muscle dissection (not even of the Wiltse plane). As a minimally invasive surgery, CBT appeared to be a viable alternative to the conventional pedicle-screw-based dynamic stabilization approach.

## Background

Various fusion techniques have been accepted as the surgical management for disc degenerative disease or spondylolisthesis, including anterior, posterior, transforaminal, and extreme lateral lumbar interbody fusion (ALIF, PLIF, TLIF and LLIF) [1–4]. In the past decade there has been an emerging option of dynamic stabilization to treat low-grade degenerative disease. The design concept of dynamic stabilization is to ameliorate the instability while maintaining segmental motility, thus yielding the potential for the prevention of adjacent segment disease (ASD). Although the actual benefit in the reduction of ASD remains elusive, more and more reports have demonstrated that dynamic stabilization is a viable option for degenerative disease or spondylolisthesis [5–7].

Pedicle-screw based dynamic stabilization systems use elastic materials, for example, a synthetic cord-and-spacer or metallic spring, instead of a rigid metallic rod, to connect between individual pedicle screws [8]. Placement of these pedicle screws requires either a midline open approach or dissection of the Wiltse plane bilaterally. Therefore, these dynamic pedicle screw stabilization systems inevitably involve extensive muscular dissection and therefore the procedure is not minimally invasive.

The authors report in this technical note an innovative method to insert four dynamic screws via a 3-cm skin incision to neutralize a degenerative spine or to stabilize low-grade spondylolisthesis. Under image guidance, the screws were placed along the cortical bone trajectory (CBT) and assembled with dynamic cords. The surgery involved microscopic decompression of the bilateral neuroforamen and dynamic screw insertion, but no discectomy (removal of ruptured part only) or interbody fusion procedures. Compared to the standard minimally invasive TLIF, the novel technique preserved the intervertebral disc and required less muscle dissection. Although the theoretical benefit of reducing ASD requires longer-term follow up to confirm, this technique spared arthrodesis and was done with minimal soft tissue or facet destruction. This is the first report to describe dynamic screw placement via the CBT.

## Methods

### Operative techniques

Four consecutive patients (2 females), with an average age of 64.75 years, who underwent minimally invasive dynamic screw stabilization with the Dynesys Dynamic Stabilization (Zimmer Biomed, Warshaw, IN, USA) system were retrospectively analyzed.

**Fig. 1:**
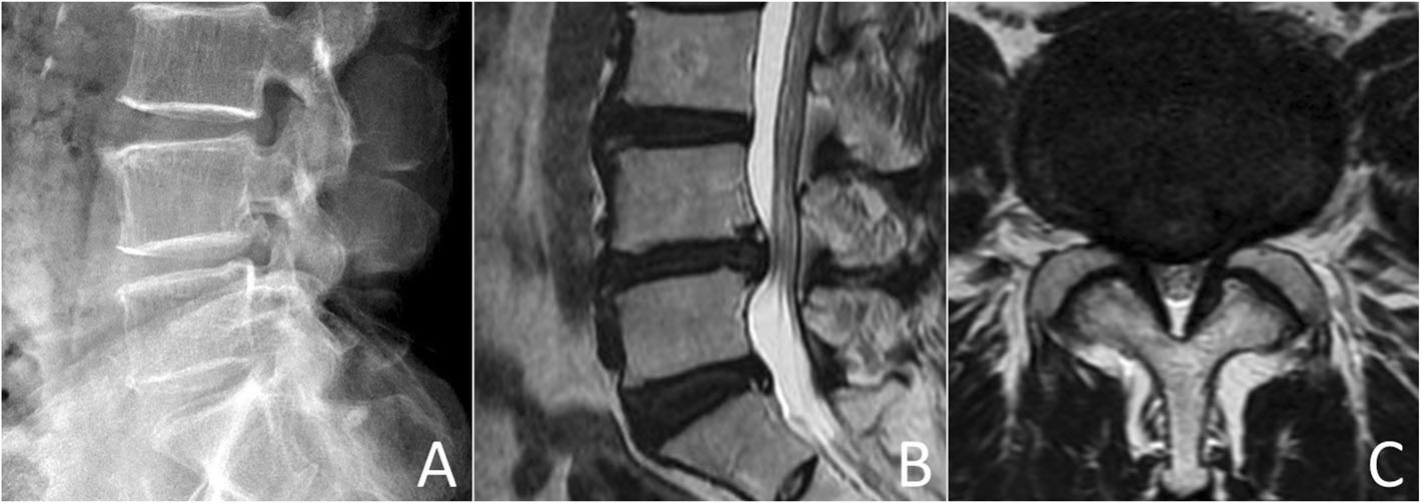
Pre-operative lateral radiograph (**a**), sagittal and axial (**b** and **c**) magnetic resonance images

After general anesthesia, the patient who had a L4–5 herniated disc with bilateral foraminostenosis (Fig. 1) was put in a prone position on a radiolucent table. The index level was identified by fluoroscopy. A midline incision, approximately 3-cm long, was made and deepened for sub-periosteum muscular dissection. Using a self-retaining blade retractor, the L4 spinous process and lamina were exposed without violation of the nearby facet joints, either L3–4 or L4–5 facets bilaterally. Under microscopes, the L4 laminectomy was carried out for resection of the hypertrophic ligamentum flavum and decompression of lateral recesses. The neuroforamen were enlarged with curved-tip Kerrison’s rongeurs and probed through with microsurgical instruments for confirmation of adequate decompression. Typically, the surgery required no discectomy. However, removal of sequestrated disc fragments was performed on those patient who had ruptured intervertebral discs.

**Fig. 2:**
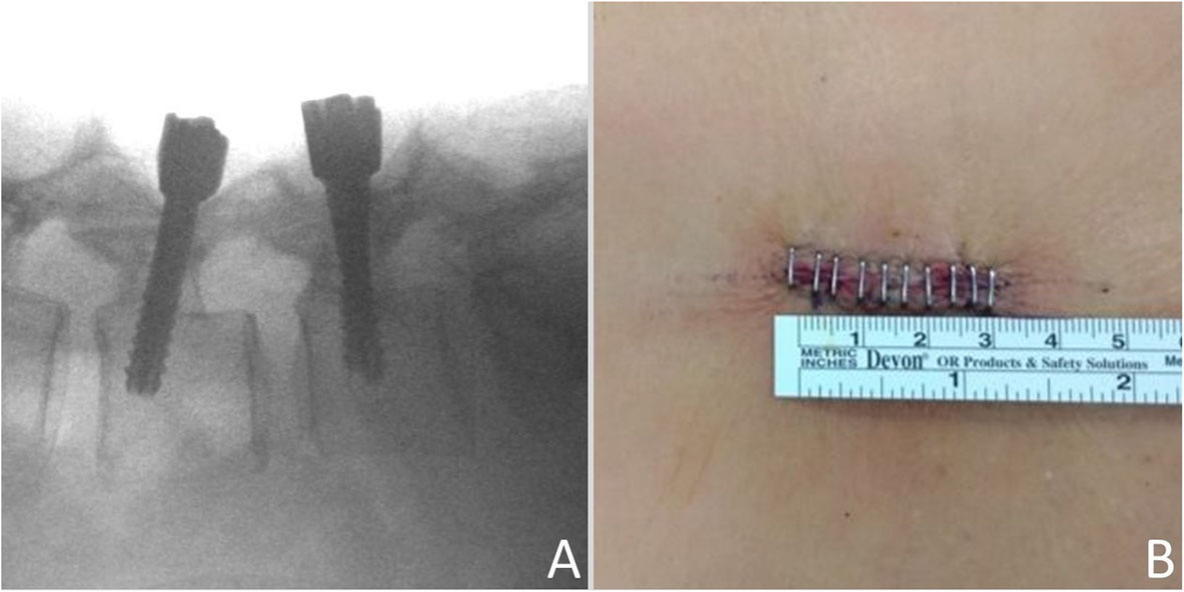
Intra-operative fluoroscopy (**a**) and post-operative incision wound measurement (**b**)

After decompression, the O-arm (Medtronic Spinal and Biologics, Memphis, TN) was brought into the room to acquire images for navigated screw placement. The reference pin was inserted into the left posterior superior iliac crest. The acquired images were transferred to the Stealth Station (Medtronic Spinal and Biologics, Memphis, TN) for spinal navigation. Prior to the beginning of instrumentation, we used an image-guided ball tip probe to confirm the accuracy of navigation and locate the entry points. The entry points of CBT screws are generally over the cephalad lateral part of the pars interarticularis, slightly caudal to the sulcus of the facet complex. An image-guided high-speed drill was used to break through the cortex. The trajectory was chosen, using the navigation system, to allow the screw to course through the dense cortical bone with the screw tip barely penetrating the cortex of the vertebral body laterally. A 5.2 mm image-guided cannulated tap was used to create the screw tract. The Dynesys screws (5.2x35mm, top loading with hydroxyapatite coating, Zimmer Biomed, Warshaw, IN) were subsequently placed under navigation. A total of four CBT screws were inserted into the L4 and L5 bodies with bi-cortical purchase (Fig. 2). Compared to traditional screw-and-rod fixation, the dynamic fixation system utilized elastic spacers (polycarbonate-urethan) and cords (polyethylene-terephthalate) to replace the rigid titanium rod in conventional constructs of instrumentation. Thus, after assembling the elastic cords and spacers and tightening of the nuts, the dynamic fixation construct remained elastic and could allow segmental motility.

## Results

### Case presentation (Table 1)

#### Case 1

A 68-year-old female presented with chronic low back pain and left sciatica for more than 2 years. The pain would be exacerbated after prolonged walking, thus limiting her activity. Her radiological evaluations demonstrated Meyerding grade one degenerative spondylolisthesis at L4–5. Prior to the visit at our institution, she had tried conservative treatment for more than 6 months. However, the pain persisted and greatly influenced her daily activity. Since she failed conservative management and her symptoms could be highly associated with the L4–5 spondylolisthesis that demonstrated instability on dynamic lateral radiographs, surgical decompression and dynamic stabilization were recommended. After surgery, her VAS of back pain was greatly improved from 5 to 3 (out of 10) and she was discharged from the hospital on post-operation day 5.

**Table 1:** Case presentation

	Age	Gender	Symptoms	Radiological diagnosis	Surgical time (mins)	Blood loss (ml)	Hospital stay (days)	Follow-up (months)	Post-operative narcotics ^a^	Pre-operative ROM	last follow up ROM ^b^	VAS improvement
Case 1	68	Female	Back pain Left sciatica	L4/5 grade I spondylolisthesis	225	250	5	28	Tramadol 75 mg/day	6.1	3.1	Back: 5- > 3 Leg: 3- > 2
Case 2	58	Male	Back pain Left sciatica	L4/5 ruptured disc	225	30	3	14	Tramadol 150 mg/day	15.4	3.1	Back:3- > 0 Leg: 10- > 0
Case 3	62	Female	Back pain	L4/5 grade I spondylolisthesis	265	100	9	15	Tramadol 37.5 mg/day	9.6	1.2	Back:9- > 2 Leg:0- > 0
Case 4	71	Male	Left Leg radiation numbness	L4/5 ruptured disc	260	50	3	31	Tramadol 150 mg/day	10.3	2.4	Back:2- > 0 Leg:1- > 0

*VAS* visual analog scale

^a^ All patients were weaned off narcotics within 3 months after operation

^b^ After dynamic stabilization, the range of motion was significantly reduced

#### Case 2

A 58-year-old male presented with low back pain and left sciatica for 6 months. The symptoms were on and off several times and it got worse prior to a visit to our clinic.

**Fig. 3:**
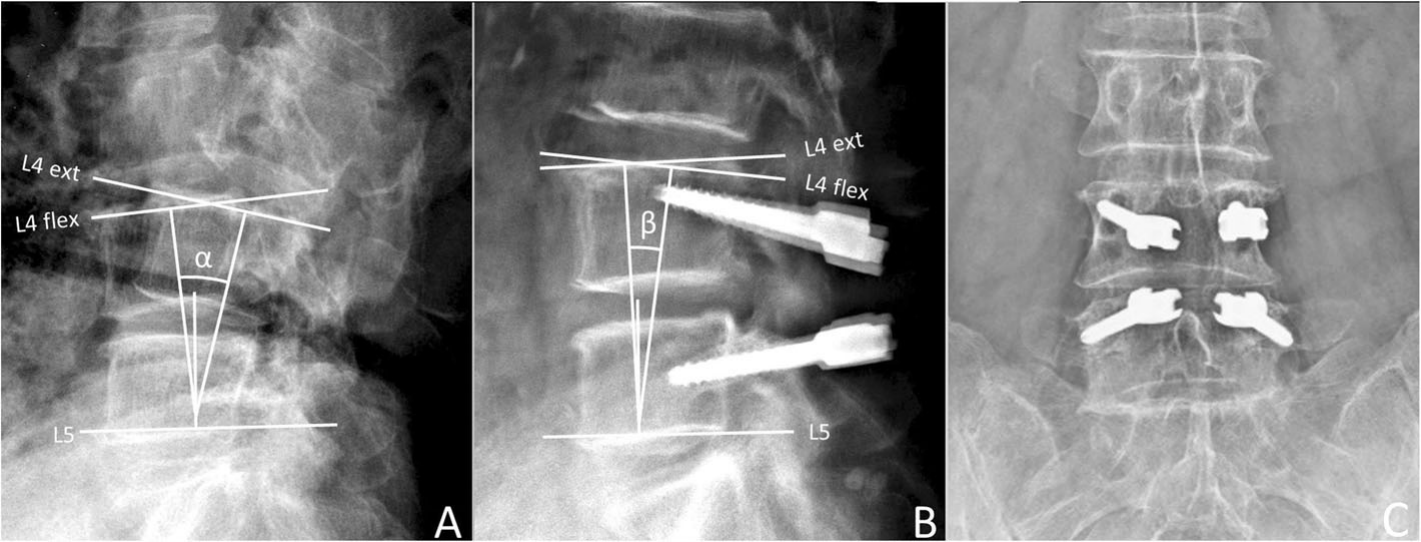
Comparison of pre-operative (**a**) and post-operative (**b**) dynamic (flexion/extension) lateral radiographs. Pre-operative ROM α:15.41°, Post-operative ROM β:3.1°. **c** Post-operative anterior-posterior fluoroscopy

His radiographic evaluations demonstrated a huge L4–5 ruptured disc with caudal migration and left L4–5 foramen compression. Since his symptoms were compatible with nerve root compression by the L4–5 ruptured disc, surgical decompression, removal of sequestrated disc fragments and dynamic stabilization were recommended. The post-operative fluoroscopy demonstrated that 3.1 degree motility was preserved at post-op 12 months (Fig. 3). Post-operation, his sciatica improved immediately. He was discharged on post-operation day 3.

#### Case 3

A 62-year-old female had a history of a traffic accident about 6 years prior to her admission and had experienced on and off low back pain since then. A second falling-down accident happened 5 years later and she experienced left sciatica, which was refractory to rehabilitation for more than 6 months. The radiographic evaluations demonstrated a herniated intervertebral disc over L4–5, grade I spondylolisthesis with severe stenosis and bilateral foraminostenosis. The patient underwent L4 laminectomy, partial removal of the ruptured disc and L4–5 dynamic stabilization. In the post-operative first few days, she still complained about back pain while it has been relieved substantially. She stayed in the hospital for another 7 days for rehabilitation.

#### Case 4

A 71-year-old male patient experienced progressive left leg radiation numbness for 1 year and his symptom was exacerbated during the 2 months prior to admission. He also had the accompanying symptom of neurologic claudication. The radiographic image revealed a L4–5 ruptured disc with caudal migration. Since his symptom was quite compatible with the L4–5 ruptured disc, the patient underwent L4 laminectomy, removal of the ruptured disc and L4–5 dynamic stabilization. Post-operatively, his radiation numbness improved gradually within the following 6 months.

## Discussion

Several clinical series have demonstrated satisfactory clinical outcomes of Dynesys dynamic stabilization (DDS) for lumbar degenerative disease [6, 9, 10]. It is still uncertain whether the claimed effects of DDS, preservation of segmental mobility and thus reduction of ASD, can be achieved and maintained in the long term [11, 12]. There also have been concerns about screw loosening, due to a lack of bone grafts that would likely fuse eventually in such dynamic constructs [7, 13, 14]. In this report the authors have described, for the first time, the innovative technique of inserting dynamic screws via a biomechanically stronger and denser corridor, the CBT. The CBT insertion provided at least two advantages for dynamic screw stabilization, solid bone-screw interface and less muscle dissection, via a mid-line to lateral insertion angle that could be done with minimal invasive surgery [15–18].

The CBT used for screw insertion was first published by Santoni et al. in 2009 for posterior midline lumbar fusion surgery. Compared with the traditional pedicle-screw trajectory used, several biomechanical studies have demonstrated the superiority of CBT screws, which could yield a 30% increase in pullout strength and a 71% increase in insertional torque [17, 18]. Moreover, using the CBT to place dynamic screws allowed avoidance of wide exposure of the facet joint and minimized muscle detachment at both the index and the cephalad facet joints. This minimally invasive approach itself might help reduce future ASD. Therefore, the CBT might be a reasonable alternative for placing dynamic screws through minimally invasive surgery. Dynamic screw stabilization via the CBT requires no discectomy or violation of any facet joints, and spares the need for muscle dissection along the Wiltse plane. Therefore, the CBT screw-based DDS is a minimally invasive option for low grade degenerative spondylolisthesis. Studies with longer term follow-up and larger sample sizes are warranted to investigate the actual clinical outcome of this novel strategy.

## Conclusion

Dynamic screw stabilization could be achieved with minimally invasive surgery via the CBT by a combination of microscopic decompression and spinal navigation. This innovative dynamic screw stabilization involves no discectomy, no interbody fusion, no facet violation, and little muscle dissection (not even of the Wiltse plane). As a minimally invasive surgery, CBT appeared to be a viable alternative to the conventional pedicle-screw-based dynamic stabilization. However, long term studies are needed to investigate such a minimally invasive surgical strategy.

## Data Availability

Not applicable.

## Abbreviations

ALIF: Anterior lumbar interbody fusion
ASD: Adjacent segment disease
CBT: Cortical bone trajectory
PLIF: Posterior lumbar interbody fusion
TLIF: Transforaminal lumbar interbody fusion
LLIF: Lateral lumbar interbody fusion

## References

[1] Mummaneni PV, Haid RW, Rodts GE (2004). Lumbar interbody fusion: state-of-the-art technical advances. Invited submission from the joint section meeting on disorders of the spine and peripheral nerves**,** March 2004. J Neurosurg Spine.

[2] Mummaneni PV, Dhall SS, Eck JC, Groff MW, Ghogawala Z, Watters WC, Dailey AT, Resnick DK, Choudhri TF, Sharan A (2014). Guideline update for the performance of fusion procedures for degenerative disease of the lumbar spine. Part 11: interbody techniques for lumbar fusion. J Neurosurg Spine.

[3] Woods KR, Billys JB, Hynes RA (2017). Technical description of oblique lateral interbody fusion at L1-L5 (OLIF25) and at L5-S1 (OLIF51) and evaluation of complication and fusion rates. Spine J.

[4] Lu G, Kuang L, Wang B. Transforaminal lumbar interbody fusion versus mini-open anterior lumbar interbody fusion with oblique self-anchored stand-alone cages for the treatment of lumbar disc herniation: a retrospective study with 2-year follow up. Spine (Phila Pa 1976). 2017;42(21):E1259–65. 10.1097/BRS.0000000000002145. 10.1097/BRS.0000000000002145 28277385

[5] Fay LY, Wu JC, Tsai TY, Wu CL, Huang WC, Cheng H (2013). Dynamic stabilization for degenerative spondylolisthesis: evaluation of radiographic and clinical outcomes. Clin Neurol Neurosurg.

[6] Kuo CH, Chang PY, Wu JC, Chang HK, Fay LY, Tu TH, Cheng H, Huang WC (2016). Dynamic stabilization for L4-5 spondylolisthesis: comparison with minimally invasive transforaminal lumbar interbody fusion with more than 2 years of follow-up. Neurosurg Focus.

[7] Wu JC, Huang WC, Tsai HW, Ko CC, Wu CL, Tu TH, Cheng H (2011). Pedicle screw loosening in dynamic stabilization: incidence, risk, and outcome in 126 patients. Neurosurg Focus.

[8] Greiner-Perth R, Sellhast N, Perler G, Dietrich D, Staub LP, Roder C (2016). Dynamic posterior stabilization for degenerative lumbar spine disease: a large consecutive case series with long-term follow-up by additional postal survey. Eur Spine J.

[9] Welch WC, Cheng BC, Awad TE, Davis R, Maxwell JH, Delamarter R, Wingate JK, Sherman J, Macenski MM (2007). Clinical outcomes of the Dynesys dynamic neutralization system: 1-year preliminary results. Neurosurg Focus.

[10] Beastall J, Karadimas E, Siddiqui M, Nicol M, Hughes J, Smith F, Wardlaw D (2007). The Dynesys lumbar spinal stabilization system: a preliminary report on positional magnetic resonance imaging findings. Spine (Phila Pa 1976).

[11] Fay LY, Chang PY, Wu JC, Huang WC, Wang CH, Tsai TY, Tu TH, Chang HK, Wu CL, Cheng H (2016). Dynesys dynamic stabilization-related facet arthrodesis. Neurosurg Focus.

[12] St-Pierre GH, Jack A, Siddiqui MM, Henderson RL, Nataraj A (2016). Nonfusion does not prevent adjacent segment disease: Dynesys long-term outcomes with minimum five-year follow-up. Spine (Phila Pa 1976).

[13] Kuo CH, Chang PY, Tu TH, Fay LY, Chang HK, Wu JC, Huang WC, Cheng H (2015). The effect of lumbar Lordosis on screw loosening in Dynesys dynamic stabilization: four-year follow-up with computed tomography. Biomed Res Int.

[14] Ko CC, Tsai HW, Huang WC, Wu JC, Chen YC, Shih YH, Chen HC, Wu CL, Cheng H (2010). Screw loosening in the Dynesys stabilization system: radiographic evidence and effect on outcomes. Neurosurg Focus.

[15] Dayani F, Chen YR, Johnson E, Deb S, Wu Y, Pham L, Singh H (2019). Minimally invasive lumbar pedicle screw fixation using cortical bone trajectory - screw accuracy, complications, and learning curve in 100 screw placements. J Clin Neurosci.

[16] Marengo N, Ajello M, Pecoraro MF, Pilloni G, Vercelli G, Cofano F, Zenga F, Ducati A, Garbossa D (2018). Cortical bone trajectory screws in posterior lumbar Interbody fusion: minimally invasive surgery for maximal muscle sparing-a prospective comparative study with the traditional open technique. Biomed Res Int.

[17] Matsukawa K, Yato Y, Kato T, Imabayashi H, Asazuma T, Nemoto K (2014). In vivo analysis of insertional torque during pedicle screwing using cortical bone trajectory technique. Spine (Phila Pa 1976).

[18] Santoni BG, Hynes RA, McGilvray KC, Rodriguez-Canessa G, Lyons AS, Henson MA, Womack WJ, Puttlitz CM (2009). Cortical bone trajectory for lumbar pedicle screws. Spine J.

